# Osseous Union of Two Teeth

**Published:** 1849-04

**Authors:** 


*Osseous Union of Two Teeth.*—Two temporary teeth, a lower central and lateral incisor, firmly united, were recently presented to us by Dr. Adair of Paris, Ky., for the Museum of the Baltimore College of Dental Surgery.—*Balt. Ed.*

